# Near-infrared fluorescent imaging techniques for the detection and preservation of parathyroid glands during endocrine surgery

**DOI:** 10.1515/iss-2021-0001

**Published:** 2021-07-30

**Authors:** Marco Stefano Demarchi, Wolfram Karenovics, Benoît Bédat, Frédéric Triponez

**Affiliations:** Department of Thoracic and Endocrine Surgery, Faculty of Medicine, University Hospitals of Geneva, Geneva, Switzerland

**Keywords:** autofluorescence, fluorescence, hypoparathyroidism, indocyanine green angiography, near-infrared-guided surgery, parathyroid glands, thyroid surgery

## Abstract

**Objectives:**

In over 30% of all thyroid surgeries, complications arise from transient and definitive hypoparathyroidism, underscoring the need for real-time identification and preservation of parathyroid glands (PGs). Here, we evaluate the promising intraoperative optical technologies available for the identification, preservation, and functional assessment of PGs to enhance endocrine surgery.

**Methods:**

We performed a review of the literature to identify published studies on fluorescence imaging in thyroid and parathyroid surgery.

**Results:**

Fluorescence imaging is a well-demonstrated approach for both *in vivo* and *in vitro* localization of specific cells or tissues, and is gaining popularity as a technique to detect PGs during endocrine surgery. Autofluorescence (AF) imaging and indocyanine green (ICG) angiography are two emerging optical techniques to improve outcomes in thyroid and parathyroid surgeries. Near-infrared-guided technology has significantly contributed to the localization of PGs, through the detection of glandular AF. Perfusion through the PGs can be visualized with ICG, which can also reveal the blood supply after dissection.

**Conclusions:**

Near infrared AF and ICG angiography, providing a valuable spatial and anatomical information, can decrease the incidence of complications in thyroid surgery.

## Introduction

More than 80,000 thyroid and parathyroid surgeries are performed yearly in the United States for the treatment of both benign and malignant thyroid diseases [1, 2]. Even for the most experienced surgeons, parathyroid glands (PGs) can be hard to distinguish from surrounding tissue due to their small size, variable position, and similarity in color to fat tissue [3, 4]. Consequently, PGs can be damaged, removed, or devascularized during endocrine surgeries, leading to postoperative complications. The most common complication resulting from inadequate PG identification and preservation is transient or definitive postoperative hypoparathyroidism, affecting 20–35 and 1–10%, respectively, of thyroidectomy patients [5, 6]. The risk of hypoparathyroidism increases in cases of total thyroidectomies, central neck dissections, and reoperative procedures [7, 8]. The low plasma levels of parathormone (PTH) associated with hypoparathyroidism can cause hypocalcemia, adversely affecting clinical outcomes and the quality of life of the patient [9]. To reduce the incidence of postoperative hypocalcemia, proper intraoperative PG identification and preservation are necessary [10, 11].

PG preservation techniques vary from basic visual inspection to highly developed optical techniques. Visual inspection is the standard procedure to identify and preserve PGs, but as the technique depends on the surgeon's experience, it is not reliable [12].

Magnification glasses with a meticulous parathyroid dissection have been proposed to prevent definitive hypoparathyroidism and also to decrease the postoperative incidence of transient hypocalcemia. However, this has not been clearly demonstrated in the literature. A systematic review and meta-analysis by Spadalis et al. state that the use of magnification techniques to identify recurrent laryngeal nerves and PGs appears to be as safe as direct vision. However, magnification techniques do not decrease the risk of recurrent laryngeal nerve injury and transient hypocalcemia after thyroid surgery compared with direct vision [13]. Moreover, in a recent article, André et al. [14] demonstrated that an active and systematic search of the parathyroid gland is not recommended due to the increased risk of gland lesion, mainly by devascularization. This has been largely studied in recent years, and the conclusion seems to be that it is best not to seek out PGs, as there is a risk of devascularization during dissection. No correlation has been shown between the number of glands detected and the risk of hypocalcemia; notably, the risks of hypocalcemia and hypoparathyroidism have been shown to increase with the number of glands visualized [10, 15], [16], [17], [18], [19], [20]. Surgeons can also intraoperatively identify PGs by sacrificing a slice of tissue for frozen section analysis or use the “float or sink” method for tissue density analysis. Both visual inspection methods are invasive, but newer identification techniques have been developed to identify PGs without damaging tissue. Near-infrared autofluorescence imaging (NIRAF) and indocyanine green (ICG) fluorescence imaging are promising optical methods for guiding PG identification and preservation [21].

Here, we present an overview of innovative near‐infrared (NIR) imaging methods for PG identification during neck endocrine surgery, evaluate their clinical results, and suggest directions for future research.

## Early techniques for PG identification

PGs are difficult to locate with the naked eye, making disruption of the PG vasculature a common complication during thyroidectomies. Disruption of the PG vasculature can cause temporary and definitive postoperative hypoparathyroidism [10, 11, 22, 23]. Therefore, there is a great demand for a reliable technique to rapidly identify PGs during surgery. Although numerous localization techniques have been evaluated, only a few intraoperative imaging modalities have been deemed effective [24].

The use of a dye or a fluorophore to visualize PGs is not new. In 1971, Dudley described the colorimetric localization of PGs with the use of intravenous methylene blue (MB) as an exogenous contrast agent [25]. Prosst et al. [26] proposed the use of aminolevulinic acid (ALA) as an alternative contrast agent, but ALA was not generally accepted due to difficulties in its application to clinical practice [27].

Rubinstein et al. introduced the first noninvasive high-resolution imaging technique, optical coherence tomography (OCT), which provides a microarchitectural characterization of structures up to 2 mm thick [28]. OCT images show characteristic features of the thyroid, parathyroid, lymph nodes, and adipose tissue, facilitating consistent identification. However, *in vivo* application trials did not achieve similar favorable results, due to technical problems and the difficulty of handling the OCT probe covered with a sterile sheath [29].

Van der Vorst et al. [21] explored the use of NIR fluorescence imaging using small, intravenous doses of MB. However, no reliable improvements in PG detection were reported. In addition, MB is potentially toxic, causing serious adverse neurological events. Therefore, no prospective randomized study has been performed with MB, and the literature has discouraged its use [30], [31], [32]. Thus, the clinical approach to parathyroid identification remained stagnant until the discovery of parathyroid autofluorescence (AF) in 2008. This discovery renewed interest in developing intraoperative PG imaging techniques that rely on the autofluorescence of parathyroid tissue in the NIR spectrum [33].

## PG identification and exogenous fluorescence imaging

Fluorescence imaging is a well-established technique used in the biomedical sciences for both *in vitro* and *in vivo* visualization of cells and tissues but has only recently been applied to surgery [34]. Fluorescence imaging leverages the property of certain substances and molecules to absorb light at a given wavelength, which briefly raises the energy of the molecule to a higher excited state. The molecule then emits light at a higher wavelength with lower energy, which can be detected and measured [34, 35]. Fluorescence imaging is conducted with exogenously administered contrast agents, such as fluorescent MB or 5-ALA [25, 32], but there is also a label‐free optical method that relies only on the intrinsic autofluorescence of certain tissues.

Hillary et al. and Tummers et al. found that fluorescent MB enabled the localization of normal PGs for up to 145 min after administrating the dye [24]. The fluorescent signal was found to be 2.6-fold higher in PGs than in thyroid tissue and 4.3-fold higher than in muscle tissue [36]. However, this approach was not further developed due to emerging concerns regarding the adverse neurotoxic effects of MB [32].

Two case reports [37, 38] and three case series [27, 39, 40] have reported the use of 5-ALA for intraoperative PG identification. ALA, a metabolic-targeting contrast agent, is the precursor of the fluorescent molecule porphyrin, which is an intermediate in the heme synthesis pathway [41]. ALA uptake increases as the number of mitochondria in PG cells increases [27]. Oral administration of 20–30 mg/kg fluorescent ALA allows detection of PG for 1–8 h. However, exposure to direct light up to 24–48 h after ALA administration causes photobleaching and many other phototoxic effects on both the skin and eyes. Additionally, the fluorescent signal is absent in some pathological PGs [27, 39].

## PG identification and autofluorescence

Until recently, fluorescence imaging techniques relied on the interaction between serum proteins and fluorescent dyes. Recently, however, the autofluorescence of multiple tissue samples has been reported. PG tissue, in particular, exhibits a unique autofluorescence signature. When the PG tissue is excited with a NIR laser at a wavelength of 785 nm, there is a spontaneous and immediate emission of fluorescent light at 820–830 nm from PGs with a 2- to 11-fold signal enhancement over that of the surrounding tissue [2]. An NIR camera or spectroscope can be used to detect autofluorescence. The detection of autofluorescence is a dye-free technique that allows noninvasive, real-time identification and precise localization of PGs. The intrinsic fluorophore responsible for this optical effect in PGs is still unknown; however, evidence suggests that it could be a calcium-sensing or a vitamin D receptor [2, 42].

Subsequent studies of thyroid surgery and PG diseases have further characterized PG autofluorescence, and have evaluated the applicability and practicality of this technique. McWade et al. and others have reported excellent PG detection rates, with a specificity of more than 80% [2, 29, 42, 43]. However, the autofluorescence signal is influenced by variables such as disease state, preoperative vitamin D levels, serum calcium levels, and body mass index [44]. To accurately identify the PGs tissue, multiple measurements are taken on each gland. A spectroscopy probe must be in contact with the tissue to make point-by-point measurements, while an optical NIR fluorescence imaging camera is contactless and provides a larger field of view. Another limitation of this technique is the difficulty of distinguishing between thyroid and parathyroid tissues. In some disease states, such as thyroiditis, the contrast in autofluorescence intensities of the two tissues may be diminished. Furthermore, brown fat, colloidal nodules, and metastatic lymph nodes can exhibit autofluorescence which may overlap with the autofluorescence from PG tissue, resulting in false positives [4, 45]. Moreover, without a standardized quantification method, the detection of an autofluorescence signal in PGs is based on the qualitative interpretation of the surgeon. Therefore, the development of devices to quantitatively detect autofluorescence in the NIR spectrum was necessary.

## NIRAF devices

Currently, there are two commercially available NIRAF devices approved by the Food and Drug Administration (FDA) which are suitable for performing the real-time identification of PG tissue during surgery: Fluobeam^®^ (Fluoptics©, Grenoble, France) and the PTeye^™^ Parathyroid Detection System (Medtronic, Inc. Minneapolis, USA previously AIBiomed Inc., Santa Barbara, CA, USA). The systems differ in the exact emitted and detected wavelength, the type of handpiece provided for detection, and the type of display used [46]. The Fluobeam^®^ system is used to explore the surgical field directly, reproducing a real‐time grayscale image through the enhancement and detection of autofluorescing tissues. Conversely, the PTeye System includes a sterile probe, which must make contact with the tissue to analyze its optical properties, producing distinct audio and visual signal.

Karl Storz and Stryker (previously Novadaq) have also developed fluorescence devices for exogenous dyes, such as ICG, but these devices are unable to detect autofluorescence (Table 1).

**Table 1:** Near-infrared autofluorescence (NIRAF) commercially available devices.

Device name	Producer	Type of display	Suited for
Fluobeam 800	(Fluoptics, Grenoble, France)	AF + ICG	Open surgery
FluobeamLX	(Fluoptics, Grenoble, France)	AF + ICG	Open surgery
PTeye	(Medtronic, Dublin, Ireland)	AF	Open surgery
PINPOINT^®^ + SPY-PHI	(Stryker, Kalamazoo, Michigan, USA)	ICG	Open surgery + laparoscopy
IMAGE1 S™ RUBINA	(Karl Storz, Tuttlingen, Germany)	ICG	Open surgery + laparoscopy
EleVision™ IR Platform	(Medtronic, Dublin, Ireland)	ICG	Open surgery + laparoscopy
Pde-neoⅡ	(HAMAMATSU PHOTONICS K.K., Systems Division, Japan)	AF + ICG	Open surgery
Quest Spectrum^®^	(Quest Medical Imaging B.V. Middenmeer, Holland)	ICG	Open surgery

AF, autofluorescence; ICG, indocyanine green.

## NIRAF in clinical practice

**Figure 1:**
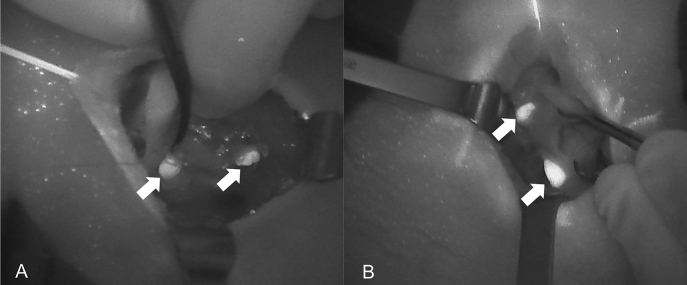
Near-infrared autofluorescence (NIRAF) images of normal parathyroid glands (PGs). Arrows indicate localization of Fluobeam LX. (A) Image of the right side after thyroidectomy. (B) Image of the right side in a case of primary hyperparathyroidism.

The introduction of NIRAF has vastly improved intraoperative imaging [47, 48]. Many recent studies (Table 2) have demonstrated that the use of NIRAF during thyroid surgery can improve surgical outcomes by facilitating PG identification (Figures 1 [Fig j_iss-2021-0001_fig_002]–3), which reduces the incidence of postoperative hypoparathyroidism. In particular, Benmilloud et al. showed that NIRAF improved PG identification and helped to reduce the rate of temporary postoperative hypocalcemia, parathyroid autotransplantation, and inadvertent parathyroid resection [48, 66]. In the literature, NIR devices have been shown to facilitate parathyroid gland identification by detecting their AF before conventional, visual recognition by the surgeon, in 37–67% of cases [49]. Additionally, these devices enable the early identification of PGs before surgical dissection, helping to preserve their vasculature [50]. A systematic review and meta-analysis by Barbieri et al. found that NIR fluorescence imaging reduced short and medium-term hypocalcemia compared to conventional surgery [51]. Moreover, NIRAF can detect subcapsular/intrathyroidal PGs or PGs that have been accidentally removed, which can then be resected from the thyroid specimen and auto planted back into the patient (Figures 4 and 5). In addition to the identification of normal PGs, NIRAF can help to identify a pathological PG. Parathyroid adenomas exhibit a more heterogeneous and less intense autofluorescence signature than that of normal PGs (Figure 6), enabling the differentiation between a normally functioning and a pathological PG [52, 53]. Furthermore, the fluorophore is resistant to freezing, heating, and fixing with formalin, meaning that the autofluorescence properties are preserved after gland resection [4, 29, 42, 43].

**Table 2:** Recent studies using near-infrared autofluorescence (NIRAF) imaging for parathyroid glands (PGs) identification during thyroidectomy.

Author, year	Study design	Sample size	Identified PGs, %	Other main findings
McWade, 2014 [42]	Case series	6	100%	NIRAF showed 2.4–8.5 times higher emission intensity from the PGs than surrounding tissue.
McWade, 2016 [44]	Clinical trial	137	97%	BMI, disease state, vitamin D, and calcium levels account significantly for variability in signal intensity. Age, gender, PTH, and ethnicity had no effect.
De Leeuw, 2016 [4]	Case series	35	98.8%	PGs fluorescence was 2.93 ± 1.59 times greater than thyroid fluorescence *in vivo*.
Falco, 2016 [45]	Case series	28	100%	NIRAF allows high rates of PG identification and is a safe, feasible, and noninvasive method for real-time intraoperative identification of PGs. No postoperative hypocalcemia or other complications related to the surgery were registered.
Kim, 2016 [73]	Case series	8	100%	PGs that were exposed or even covered by connective tissues or blood vessels could be detected with strong emission.
Falco, 2017 [74]	Case series	74	100%	The number of PGs identified was significantly increased by the use of NIRAF. The differences in fluorescent intensity among PGs, thyroid glands, and background were not affected by age, sex, and histopathological diagnosis.
Ladurner, 2017 [75]	Prospective trial	30	80.9%	NIRAF can be used to distinguish PGs from other cervical tissues. There were no noticeable differences between parathyroid adenomas, hyperplasia, and normal PGs.
Kahramangil, 2017 [76]	Clinical trial	22	98%	Autofluorescence detects more frequently PGs before recognition with the naked eye compared to indocyanine green fluorescence. No differences in postoperative hypocalcemia were detected.
Kahramangil, 2018 [52]	Retrospective cohort	210	98%	NIRAF facilitated PG identification before conventional recognition by the surgeon, 37–67% of the time. NIRAF alongside conventional visual cues to aid identification of PGs during neck operations.
Ladurner, 2018 [77]	Case series	20	90.2%	Neither lymph nodes nor thyroid revealed substantial autofluorescence and nor did adipose tissue NIRAF can be used to identify and preserve PGs during thyroidectomy.
Benmiloud, 2018 [78]	Before and after controlled study	93	76.3%	NIRAF reduced postoperative hypocalcemia and PGs autotransplantation rate.
Kim, 2018 [50]	Prospective trial	38	92.8%	NIRAF PG mapping has an excellent accuracy rate. This technique may be helpful for the early identification of PGs during thyroidectomy.
Alesina, 2018 [69]	Prospective trial	5	68.8%	NIRAF allows for enhanced visualization of the parathyroid tissue during video-assisted thyroidectomy with neither intraoperative nor postoperative complications.
DiMarco, 2019 [79]	Prospective cohort	269	85.8%	NIRAF doesn't reduce the incidence of missed inadvertent parathyroidectomy. There was no significant difference in serum calcium or PTH between NIRAF and control groups.
Dip, 2019 [80]	Randomized controlled trial	170	NR (increased from a mean of 2.6–3.5)	NIRAF increases intraoperative identification of PGs and decreases the incidence of postoperative hypocalcemia.
Benmiloud, 2019 [60]	Randomized clinical trial	241	75.9%	NIRAF increases parathyroid preservation after total thyroidectomy and helps to improve the early postoperative hypocalcemia rate significantly.

PGs, parathyroid glands; NIRAF, near-infrared autofluorescence; BMI, body mass index; PTH, parathyroid hormone; NR, not reported.

**Figure 2: j_iss-2021-0001_fig_002:**
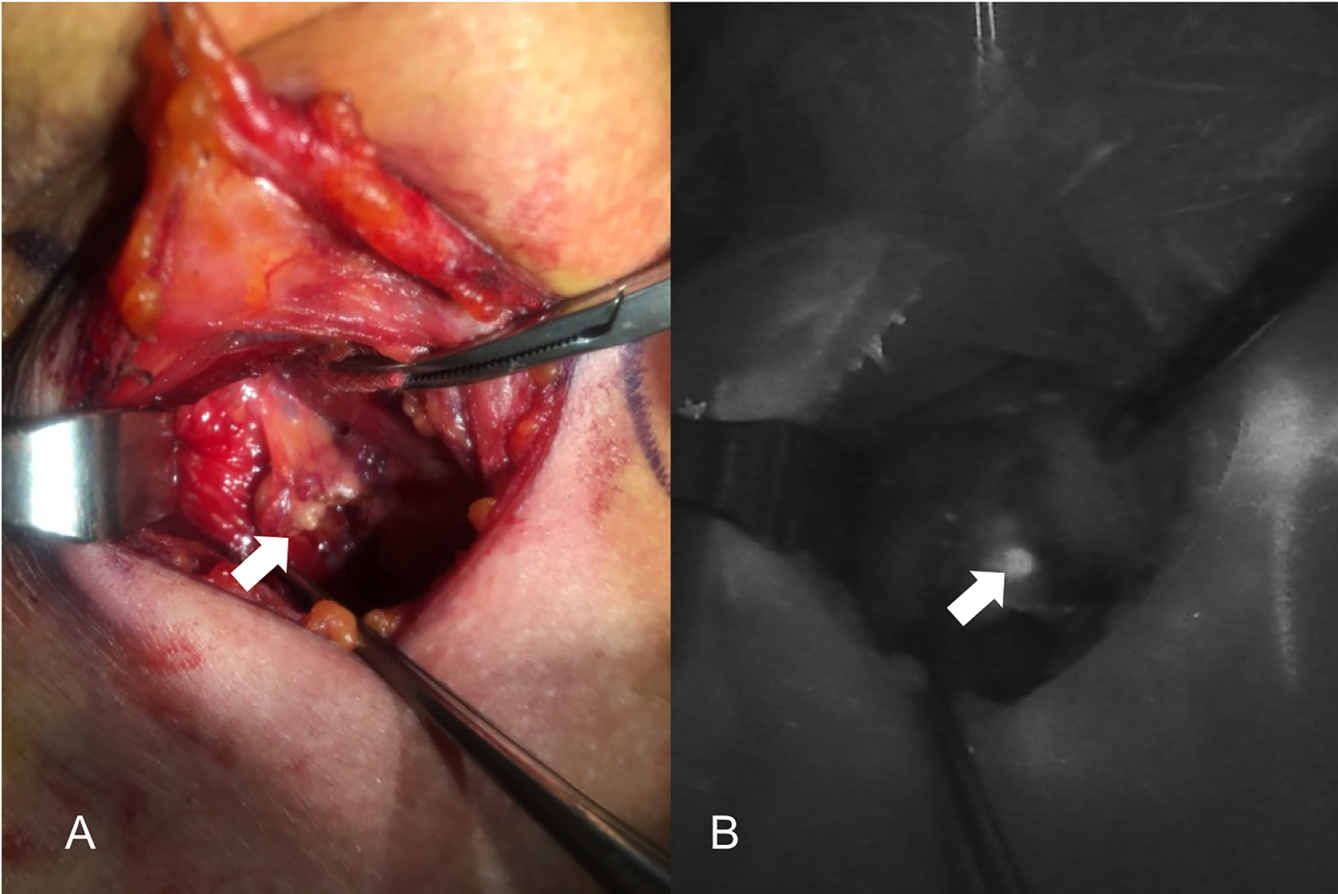
Comparison of unaided visualization (A) and near-infrared autofluorescence (NIRAF) imaging using Fluobeam LX (B) of the left side superior parathyroid. The parathyroid gland (PG) is indicated with the white arrow.

**Figure 3:**
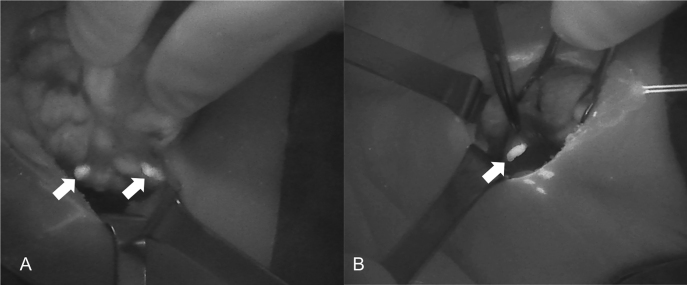
Near-infrared autofluorescence (NIRAF) images using Fluobeam LX showing the autofluorescence of the parathyroid glands (PGs). (A) Two PGs after superior pole dissection and the medialization of the right thyroid lobe. (B) Superior PG after medialization of the left thyroid lobe. PGs are indicated with white arrows.

**Figure 4:**
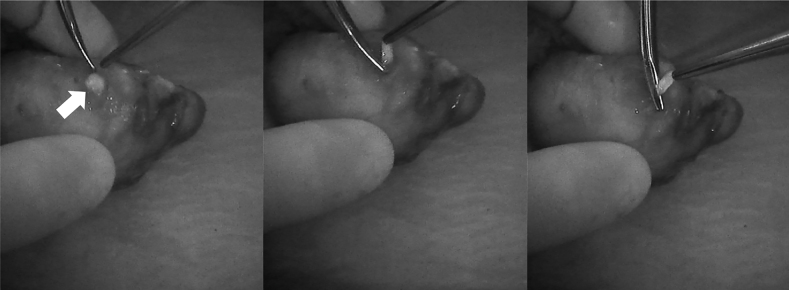
Three phases of dissection of subcapsular parathyroid (indicated with a white arrow), which was detected using Fluobeam LX near-infrared autofluorescence (NIRAF) imaging. The parathyroid gland (PG) can then be transplanted.

**Figure 5:**
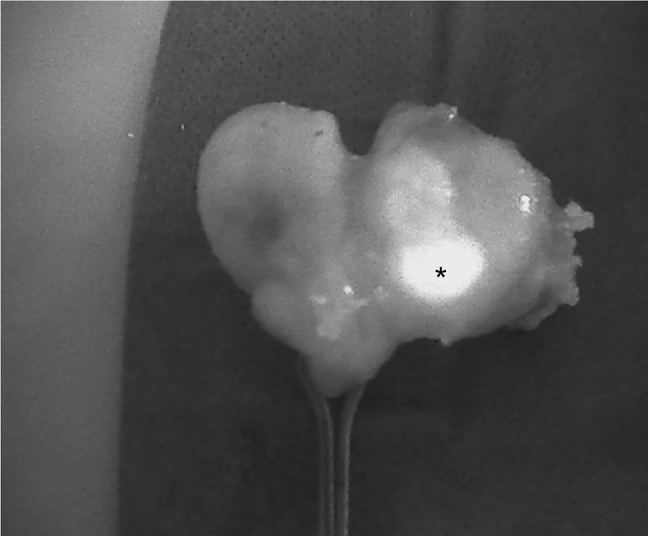
An intrathyroidal parathyroid gland (PG) (indicated with *) detected using Fluobeam LX near-infrared autofluorescence (NIRAF) imaging. The PG can be dissected and transplanted.

**Figure 6:**
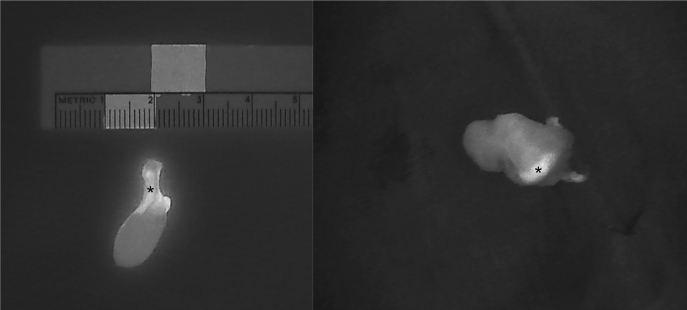
Intraoperative near-infrared autofluorescence (NIRAF) images of parathyroid adenomas after resection, demonstrating the heterogeneous and less intense fluorescence pattern that differentiates diseased parathyroid glands (PGs) from normal PGs (indicated with *). Frequently, the most intense NIRAF signal comes from residual normal parathyroid tissue in the adenoma.

However, the perfusion status and viability of the PG tissues cannot be assessed on the basis of this single parameter. Considering the importance of PG vitality in thyroid surgery, a method for the confirmation of vascular integrity is also needed.

## PG identification and ICG angiography

ICG is an FDA-approved fluorescent contrast agent which has been used since 1956, and, when systemically administered, has few side effects and low toxicity [43]. ICG fluorescence imaging enables the real‐time direct imaging and assessment of tissue perfusion and vascularization, and as a result, it has become one of the most common imaging techniques. ICG is excited at 780–805 nm and emits a maximum NIR signal at 830–835 nm [34, 54]. IGC is an amphiphilic tricarbocyanine dye that travels through the circulatory system, has a half-life of approximately 3–5 min, and is excreted in 15–20 min by the biliary system [55]. For visualization, a dose of 0.2–0.5 mg/kg is recommended and can be repeated as required, as long as the daily dose does not exceed 5 mg/kg [55, 56]. Approximately 30 s to 2 min after injection, NIR fluorescence camera images can capture the distribution of ICG in the explored tissue. ICG was first used in ophthalmology for the detection of macular degeneration and retinal angiography [34, 35]. Soon after, this technique was applied to colorectal surgeries for the evaluation of intestinal anastomosis by imaging the intestinal microvasculature [57], [58], [59].

**Figure 7:**
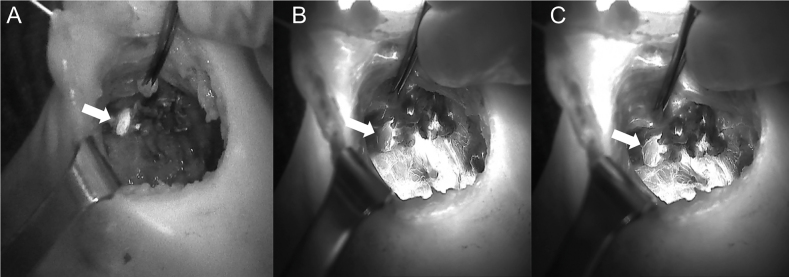
A sequence of near-infrared autofluorescence (NIRAF) images showing a parathyroid gland (PG), indicated with a white arrow. (A) Autofluorescence of the PG prior to injection of indocyanine green (ICG). (B) and (C) Diffusion of the ICG contrast agent, showing a well-vascularized PG.

**Figure 8:**
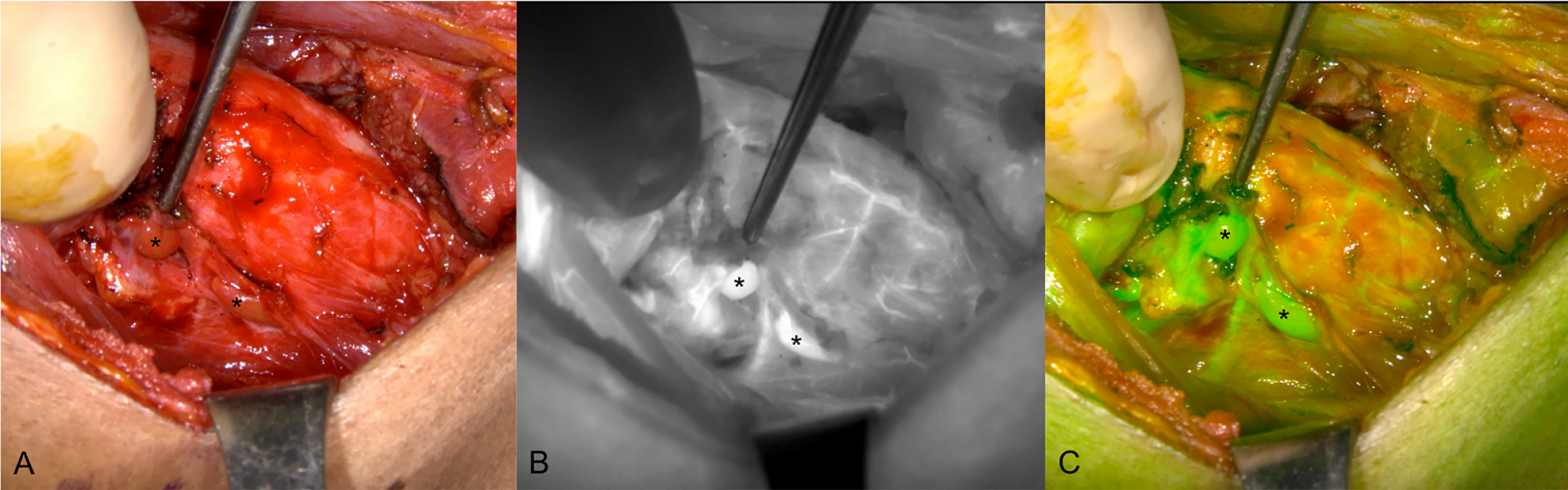
Indocyanine green (ICG) angiography images of the right side parathyroid glands (PGs) (indicated with *) after thyroidectomy surgery. (A) Unaided visualization of the tissue. (B) Near-infrared autofluorescence (NIRAF) grayscale image and (C) Green fused image showing two well-vascularized PGs after injection of ICG.

Most recently, ICG has been proposed to be the most suitable agent for the intraoperative assessment of the PG vascularization, which is closely correlated with parathyroid function (Figures 7 and 8) [56, 60], [61], [62], [63], [64]. Many studies have evaluated the use of ICG angiography to identify normal PGs and assess their perfusion in thyroidectomies (Table 3). These studies suggest that ICG angiography is a safe, feasible, effective, and easy technique to identify and preserve PGs [65]. Owing to the contribution of ICG angiography to intraoperative decision-making, the technique may be widely used in the future, especially as new applications are developed. For example, the technique is being developed for mini-invasive and robotic surgery applications, including the transoral endoscopic thyroidectomy vestibular approach and the robotic bilateral axillo‐breast approach [66], although studies are limited.

**Table 3:** Recent studies using indocyanine green (ICG) angiography for parathyroid glands (PGs) identification and perfusion during thyroid surgery.

Author, year	Study design	Sample size	ICG dose	PGs perfusion (score)	Identified PGs, %	Other main findings
Zaidi, 2016 [71]	Prospective trial	27	5 mg	Qualitative (0–3)	83.5%	PTH levels in postoperative day one correlated with PGs fluorescence signal.
Lang, 2017 [67]	Case series	94	2.5 mg	Quantitative (PG/trachea ratio)	95.3%	PTH levels were normal and correlated with PGs fluorescence signal.
Yu, 2017 [81]	Prospective cohort	22	10 mg	ND	97%	The ICG group had a significantly lower rate of incidental parathyroidectomy than the control group.
Vidal Fortuny, 2016 [82]	Prospective cohort	36	8.75 mg	Qualitative (0–2)	91.9%	PTH was normal for all patients with at least one well-vascularized PG.
Alesina, 2018 [69]	Prospective trial	5	7.5 mg	Visual estimation	75%	ICG injection has been used for guiding the dissection of the gland in three cases and for confirmation of the vascular supply at the end of the procedure in two cases PTH was normal in postoperative day one in all patients.
Vidal Fortuny, 2018 [61]	Clinical trial	196	8.75 mg	Qualitative (0–2)	77.6%	ICG angiography reliably predicts the vascularization of the PGs one well-perfused PG using ICG angiography is a reliable predictor of the absence of postoperative hypoparathyroidism.
Rudin, 2019 [62]	Retrospective study	210	10 mL	Qualitative (0–2)	81.7%	ICG angiography can guide autotransplantation. At least two vascularized glands on ICGA may predict postoperative PG function. Transient hypoparathyroidism was present in 37% of patients without difference between the ICG group and control.
Razavi, 2019 [70]	Retrospective cohort	111	5 mg	Qualitative (0–2)	ND	No significant difference was found in PTH levels, symptomatic hypocalcemia, or length of stay with vs. without ICG. Low‐flow ICG patterns may lead to unnecessary parathyroid autotransplantation.
Gálvez‐Pastor, 2019 [68]	Prospective cohort	39	5 mg	4-ICG score	82%	The 4‐ICG score showed good discrimination in terms of predicting postoperative hypocalcemia.
Jin, 2019 [65]	Case series	26	5 mg	Qualitative (0–3)	100%	With an ICG score of 2, postoperative PTH levels were in the normal range.
van den Bos, 2019 [83]	Case series	26	7.5 mg	Qualitative (1–3)	43%	The use of ICG can provide more certainty about the location of the PGs.

ICG, indocyanine green; PGs, parathyroid glands; PTH, parathyroid hormone; ND, not defined.

Studies suggest that using ICG angiography can help preserve at least one well‐vascularized PG after total thyroidectomy. Normal PTH levels have been recorded on the first postoperative day, which indicates a 100% of positive predictive value for excluding postoperative hypoparathyroidism [63, 65, 67], [68], [69]. However, some studies have shown less positive results for the prediction of parathyroid function [62, 70]. This difference is likely related to the individual visual interpretation of the grayscale images produced during angiography, which lack standardized numerical criteria [56]. In clinical practice, the vascularization of PGs can be evaluated either by quantitative analysis of the ICG fluorescence signal [67] or by qualitative estimation of dye uptake, which depends on the surgeon's judgment [56, 61, 71]. To date, quantification using ICG imaging is only possible in post-processing and not in real-time. Still, the available literature suggests that PG function resumes in patients with moderately well-vascularized PGs.

One of the challenges of this technique is that even the most experienced surgeon may confuse the PGs with other anatomical structures, such as the thyroid, thymus nodules, or lymph nodes [72]. In such cases, the assessment of good vascularization of what is actually non-PG tissue could lead to false assumptions about the risk of developing postoperative hypoparathyroidism. Thus, ICG angiography is currently only used to evaluate PGs after surgical resection. While there are many promising clinical trials aimed at reducing the extent of PG resection and the incidence of post-thyroidectomy hypoparathyroidism using ICG angiography [64], further studies are needed.

## Conclusion

Although most thyroid and parathyroid operations are not complicated, difficulties in accurately locating PGs during thyroid surgery may result in the inadvertent resection of PG and the disruption of the PG vasculature, causing postoperative hypoparathyroidism. It is too early to assume that NIRAF imaging and ICG angiography will have a major role in the intraoperative identification and preservation of PGs, but both modalities have provided valuable spatial and anatomical information. The techniques can be applied easily during surgery, enabling the real‐time detection of both healthy and diseased PGs, and have reliably and substantially decreased the incidence of complications in thyroid surgery. However, it is important to understand the limitations of these techniques and to improve their surgical applications. Therefore, further studies, including randomized controlled trials and meta-analyses, are needed.

## Supplementary Material

Supplementary Material
