# Supplementary material for: Near-infrared fluorescent imaging techniques for the detection and preservation of parathyroid glands during endocrine surgery
Source: Innov Surg Sci. 2021 Jul 30;7(3-4):87–98. doi: 10.1515/iss-2021-0001 (PMC9742281; doi:10.1515/iss-2021-0001)
Supplement: Supplementary file 1 — Supplementary Material [file j_iss-2021-0001_suppl.pdf]

Reviewer Assessment

M S Demarchi et al.: Near-infrared fluorescent imaging techniques for the detection and preservation of parathyroid glands during endocrine surgery

Reviewers' Comments to Original Submission

Reviewer 1: anonymous

Date received: 18-Jan-2021
Reviewer recommendation: **Return to author for major modifications**
Reviewer overall scoring: **Medium**

Assessment Form scores: 5 = High/Yes; 3 = Medium/Adequate; 1 = Low

Is the subject area appropriate for the journal	5				
Does the title clearly reflect the paper's content?			3		
Does the abstract clearly reflect the paper's content	5				
Do the keywords clearly reflect the paper's content?	5				
Does the introduction present the problem clearly?	5				
Are the results/ conclusions justified?			3		
How comprehensive and up-to-date is the subject matter presented?		4			
How adequate is the data presentation?			3		
Are units and terminology used correctly?	5				
Is the number of cases adequate?	5				
Are the experimental methods/ clinical studies adequate?	5				
Is the length appropriate in relation to the content?	5				
Does the reader get new insights from the article?		4			
Please rate the practical significance.				2	
Please rate the accuracy of methods.				2	
Please rate the statistical evaluation and quality control.				2	
Please rate the appropriateness of the figures and tables.			3		
Please rate the appropriateness of the references.	5				
Please evaluate the writing style and use of language.	5				
Please judge the overall scientific quality of the manuscript.			3		
Are the methods used worthy of reproduction in greater deal?	No				
Would you be willing to review a revision of this manuscript?	Yes				

Comments to author: The paper deals with a new and interesting technical approach to parathyroid gland identification and preservation aiming to reduce the relatively high frequency of transient and further reduce the already low frequency of permanent hypoparathyroidism after total thyroidectomy. Strength of the paper is the historical introduction and methodological descriptions of techniques. Weaknesses are the report of current data concerning the net benefit of techniques on the improvement of postop transient and permanent hypopara rates after thyroidectomy due to benign nodular goiter, autoimmune thyroid disease, or thyroid cancer.

Comments:

1. Most surgeons today are using magnifying glasses enabling not only to perform meticulous capsular dissection thyroidectomy but at the same time to identify correctly parathyroid glands at their typical position, and to trace the vessels supplying the glands. Four-gland-identification by magnifying glasses therefore may be regarded as the "gold standard" of conventional thyroidectomy. The authors should review and discuss this already well established technique in the light of new visualzation techniques described in the overview.

2. Soon after first descriptions of NIRAF the question arose to which extent the technique may contribute to significant reduction of transient but in particular to permanent hypopara rates after total thyroidectomy for nodular goiter, autoimmune thyroid disease, and thyroid cancer, especially as far as central node dissection is performed in addition to total thyroidectomy for the latter. Authors neither did show current data of net benefit of NIRAF and ICG nor discussed that issue on the background of thyroid diseases as mentioned.

3. Tables 2 and 3 instead of "identified PGs (%)" should indicate the numbers of PG, or how often at least 3 or 4 PGs have been identified in the studies listed. Moreover, it should be stated per study listed, which cohort of patients in respect to underlying thyroid disease have been analyzed, and which surgical approach, thyroidectomy without or with magnifying glasses, have been used for control.

Reviewer 2: : anonymous

Date received: 15-Mar-2021

Reviewer recommendation: **Accept in present form**

Reviewer overall scoring: **High**

Assessment Form scores: 5 = High/Yes; 3 = Medium/Adequate; 1 = Low

Is the subject area appropriate for the journal	5				
Does the title clearly reflect the paper's content?		4			
Does the abstract clearly reflect the paper's content	5				
Do the keywords clearly reflect the paper's content?	5				
Does the introduction present the problem clearly?	5				
Are the results/ conclusions justified?	5				
How comprehensive and up-to-date is the subject matter presented?			3		
How adequate is the data presentation?	5				
Are units and terminology used correctly?	5				
Is the number of cases adequate?	5				
Are the experimental methods/ clinical studies adequate?	5				
Is the length appropriate in relation to the content?	5				
Does the reader get new insights from the article?			3		
Please rate the practical significance.		4			

Please rate the accuracy of methods.		4			
Please rate the statistical evaluation and quality control.	5				
Please rate the appropriateness of the figures and tables.	5				
Please rate the appropriateness of the references.	5				
Please evaluate the writing style and use of language.	5				
Please judge the overall scientific quality of the manuscript.		4			
Are the methods used worthy of reproduction in greater deal?	Yes				
Would you be willing to review a revision of this manuscript?					

Comments to author: I have one minor remark. Despite that I estimate your publication as good, worthy and up to date knowing of the high percentage of postoperative hypoparathyreoidism and its long-term consequences.
In the text section regarding "Early techniques for PG identification" I recommend to classify the presented methods in timeline for better understanding.

Authors' Response to Reviewer Comments

Date received: 24-Mar-2021

Response to reviewer 1

The paper deals with a new and interesting technical approach to parathyroid gland identification and preservation aiming to reduce the relatively high frequency of transient and further reduce the already low frequency of permanent hypoparathyroidism after total thyroidectomy. Strengst of the paper is the historical introduction and methodological descriptions of techniques. Weaknesses are the report of current data concerning the net benefit of techniques on the improvement of postop transient and permanent hypopara rates after thyroidectomy due to benign nodular goiter, autoimmune thyroid disease, or thyroid cancer.

We are grateful to the reviewer for the consideration of our article. Indeed, these comments are very pertinent to improve the quality of the paper. Therefore, we extended the part concerning the current data on the benefit of fluorescence techniques as follows: "In the literature, near infrared devices have been shown to facilitate parathyroid gland identification by detecting their AF before conventional, visual recognition by the surgeon, in 37–67% of cases [49]. Additionally, these devices enable the early identification of parathyroid glands before surgical dissection, helping to preserve their vasculature [50]. A systematic review and meta-analysis by Barbieri et al. found that near-infrared fluorescence imaging reduced short and medium term hypocalcemia compared to conventional surgery [51]."

A manuscript with tracked changes will be provided to facilitate identifications of the various modifications and corrections.

1. Most surgeons today are using magnifying glasses enabling not only to perform meticulous capsular dissection thyroidectomy but at the same time to identify correctly parathyroid glands at their typical position, and to trace the vessels supplying the glands. Four-gland-identification by magnifying glasses therefore may be regarded as the "gold standard" of conventional thyroidectomy. The authors should review and discuss this already well-established technique in the light of new visualzation techniques described in the overview.

Thank you for this remarkable comment. We added in the text a part concerning the use of magnification glasses and the four-gland identification.

Effectively magnification and a meticulous parathyroid dissection has been proposed as a factor that could contribute to preventing definitive hypoparathyroidism and also to decrease the postoperative incidence of transient hypocalcaemia but this hasn't been clearly demonstrated in literature to our knowledge. We added as suggested this element in the text as followed:

"Magnification glasses with a meticulous parathyroid dissection has been proposed to prevent definitive hypoparathyroidism and also to decrease the postoperative incidence of transient hypocalcaemia. However, this has not been clearly demonstrated in the literature. A systematic review and meta-analysis by Spadalis et al. states that the use of magnification techniques to identify recurrent laryngeal nerves and parathyroid glands appears to be as safe as direct vision. However, magnification techniques do not decrease the risk of recurrent laryngeal nerve injury and transient hypocalcemia after thyroid surgery compared with direct vision [13]. Moreover, in a recent article, André N. et al. [14] demonstrated that active and systematic search of the parathyroid gland is not recommended due to the increased risk of gland lesion, mainly by devascularization. This has been largely studied in recent years, and the conclusion seems to be that it is best not to seek out parathyroid glands, as there is a risk of devascularization during dissection. No correlation has been shown between the number of glands detected and the risk of hypocalcemia; notably, the risks of hypocalcemia and hypoparathyroidism have been shown to increase with the number of glands visualized [10, 15–20]." [...]

"In the literature, near infrared devices have been shown to facilitate parathyroid gland identification by detecting their AF before conventional, visual recognition by the surgeon, in 37–67% of cases [49]. Additionally, these devices enable the early identification of parathyroid glands before surgical dissection, helping to preserve their vasculature [50]. A systematic review and meta-analysis by Barbieri et al. found that near-infrared fluorescence imaging reduced short and medium term hypocalcemia compared to conventional surgery [51].]"

2. Soon after first descriptions of NIRAF the question arose to which extent the technique may contribute to significant reduction of transient but in particular to permanent hypopara rates after total thyroidectomy for nodular goiter, autoimmune thyroid disease, and thyroid cancer, especially as far as central node dissection is performed in addition to total thyroidectomy for the latter. Authors neither did show current data of net benefit of NIRAF and ICG nor discussed that issue on the background of thyroid diseases as mentioned.

Thank you for this comment.

In the section "NIRAF in clinical practice" we present the benefits of NIRAF in clinical practice and in the "PG identification and ICG angiography" we present current data and benefits of the use of ICG angiography. We added a new section with the latest reviews and articles on the subject as previously mentioned.

Actual literature doesn't yet make a clear distinction in the mentioned pathological categories.

3. Tables 2 and 3 instead of "identified PGs (%)" should indicate the numbers of PG, or how often at least 3 or 4 PGs have been identified in the studies listed. Moreover, it should be stated per study listed, which cohort of patients in respect to underlying thyroid disease have been analyzed, and which surgical approach, thyroidectomy without or with magnifying glasses, have been used for control.

The shape of these tables has been made in order to uniform data and since it is a review of literature the number of 3/4 PGs is frequently not mentioned. Pathological categories or the use of magnification glasses is an information rarely mentioned in the listed articles moreover as above mentioned a Systematic Review and Meta-Analysis of Spadalis et Al. states that the use of magnification techniques for identification of RLN and parathyroid glands seems to be as safe as direct vision concerning postoperative hypoparathyroidism.

Response to reviewer 2

I have one minor remark. Despite that I estimate your publication as good, worthy and up to date knowing of the high percentage of postoperative hypoparathyreoidism and its long-term consequences.
In the text section regarding "Early techniques for PG identification" I recommend to classify the presented methods in timeline for better understanding.

We are grateful to the reviewer for the consideration of our article. Indeed, this comment will improve the quality of the paper. We have changed as asked the timeline of the presented methods as the different techniques has been proposed.

Reviewers' Comments to Revised Submission

Reviewer 1: anonymous

Date received: 13-Apr-2021
Reviewer recommendation: **Accept in present form**
Reviewer overall scoring: **High**

Assessment Form scores: 5 = High/Yes; 3 = Medium/Adequate; 1 = Low

Is the subject area appropriate for the journal	5				
Does the title clearly reflect the paper's content?			3		
Does the abstract clearly reflect the paper's content	5				
Do the keywords clearly reflect the paper's content?	5				
Does the introduction present the problem clearly?	5				
Are the results/ conclusions justified?		4			
How comprehensive and up-to-date is the subject matter presented?	5				
How adequate is the data presentation?	5				
Are units and terminology used correctly?	5				
Is the number of cases adequate?	5				
Are the experimental methods/ clinical studies adequate?	5				
Is the length appropriate in relation to the content?	5				
Does the reader get new insights from the article?	5				
Please rate the practical significance.			3		
Please rate the accuracy of methods.		4			
Please rate the statistical evaluation and quality control.		4			
Please rate the appropriateness of the figures and tables.		4			
Please rate the appropriateness of the references.	5				
Please evaluate the writing style and use of language.	5				
Please judge the overall scientific quality of the manuscript.		4			
Are the methods used worthy of reproduction in greater deal?	Yes				
Would you be willing to review a revision of this manuscript?	No				

Comments to author: No further comments.

Reviewer 2: anonymous

Date received: 14-Apr-2021
Reviewer recommendation: **Accept in present form**
Reviewer overall scoring: **Excellent**

Assessment Form scores: 5 = High/Yes; 3 = Medium/Adequate; 1 = Low

Is the subject area appropriate for the journal	5				
Does the title clearly reflect the paper's content?	5				
Does the abstract clearly reflect the paper's content	5				
Do the keywords clearly reflect the paper's content?	5				
Does the introduction present the problem clearly?	5				
Are the results/ conclusions justified?	5				
How comprehensive and up-to-date is the subject matter presented?		4			
How adequate is the data presentation?	5				
Are units and terminology used correctly?	5				
Is the number of cases adequate?	5				
Are the experimental methods/ clinical studies adequate?	5				
Is the length appropriate in relation to the content?	5				
Does the reader get new insights from the article?	5				
Please rate the practical significance.	5				
Please rate the accuracy of methods.	5				
Please rate the statistical evaluation and quality control.	5				
Please rate the appropriateness of the figures and tables.	5				
Please rate the appropriateness of the references.	5				
Please evaluate the writing style and use of language.	5				
Please judge the overall scientific quality of the manuscript.	5				
Are the methods used worthy of reproduction in greater deal?					
Would you be willing to review a revision of this manuscript?	Yes				

Comments to author: none
